# Missed Distal Femoral Cortical Perforation During Intramedullary Nailing of an Intertrochanteric Femur Fracture: The Role of Early Advanced Imaging

**DOI:** 10.7759/cureus.74310

**Published:** 2024-11-23

**Authors:** Yekeen Abu-Shiraz, Matthew S Smith, Joshua Bryan, Madana Mohana Reddy Vallem, Sreenivasulu Metikala, Khalid Hasan

**Affiliations:** 1 Medicine, Virginia Commonwealth University School of Medicine, Richmond, USA; 2 Orthopaedic Surgery, Virginia Commonwealth University, Richmond, USA; 3 Orthopaedics, Virginia Commonwealth University, Richmond, USA; 4 Orthopaedics, Virginia Commonwealth University School of Medicine, Richmond, USA

**Keywords:** anterior cortical perforation, femur intertrochanteric fracture, fragility hip fracture, role of early advanced imaging, sclerotic bone lesions

## Abstract

Distal femoral anterior cortical perforation is a rare complication of intramedullary nailing for proximal femur fractures. Awareness and intraoperative preventive measures are key to minimizing the risk of this complication. We report a case of a patient who experienced an anterior cortical breach of the distal femur during routine antegrade nailing for an intertrochanteric fracture, which was attributed to a sclerotic lesion in the distal femur. Additionally, we describe the management of this complication, along with an analysis of risk factors and effective prevention strategies.

## Introduction

Proximal femur fractures are common musculoskeletal injuries in the elderly, typically resulting from ground-level falls or low-energy trauma [1]. In clinical practice, intramedullary (IM) nailing has become the standard treatment modality due to the stability of the construct, which allows for early mobilization and weight bearing while the fracture heals [2]. Historically, long femoral nails have been recommended for unstable fractures and patients with osteoporotic bones, given their high risk of falls. However, newer studies demonstrate equivalent results with shorter nails [3]. Distal femoral anterior cortical perforation (DFACP) is an uncommon complication of long IM nailing for proximal femur fractures, with potentially serious consequences if not recognized and addressed promptly. This article details an intraoperative DFACP that occurred in an elderly woman during a routine IM nailing procedure for an acute intertrochanteric fracture. The complication was attributed to a bony infarct in the distal femoral medullary canal, highlighting the critical role of intraoperative biplanar fluoroscopy in assessing the entire length of the intended implant trajectory. Within this case report, we outline our approach to managing this complication while reviewing the associated risk factors and effective prevention strategies.

## Case presentation

**Figure 1:**
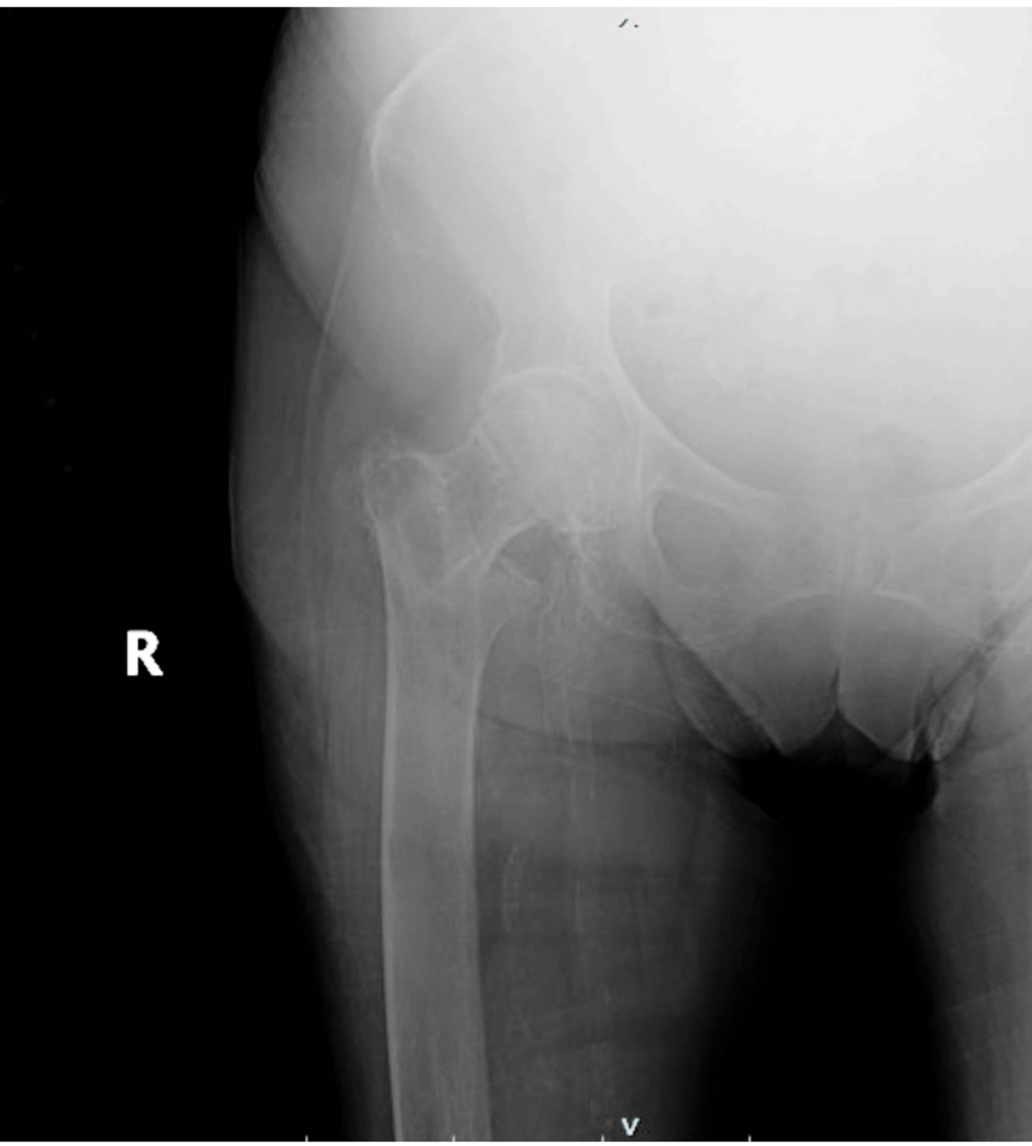
Initial injury anteroposterior (AP) radiograph showing an intertrochanteric fracture of the right hip with generalized osteopenia

A 91-year-old female with a history of dementia, chronic obstructive pulmonary disease, and peripheral arterial disease experienced a ground-level fall, resulting in a right intertrochanteric proximal femur fracture (Figure 1).

**Figure 2:**
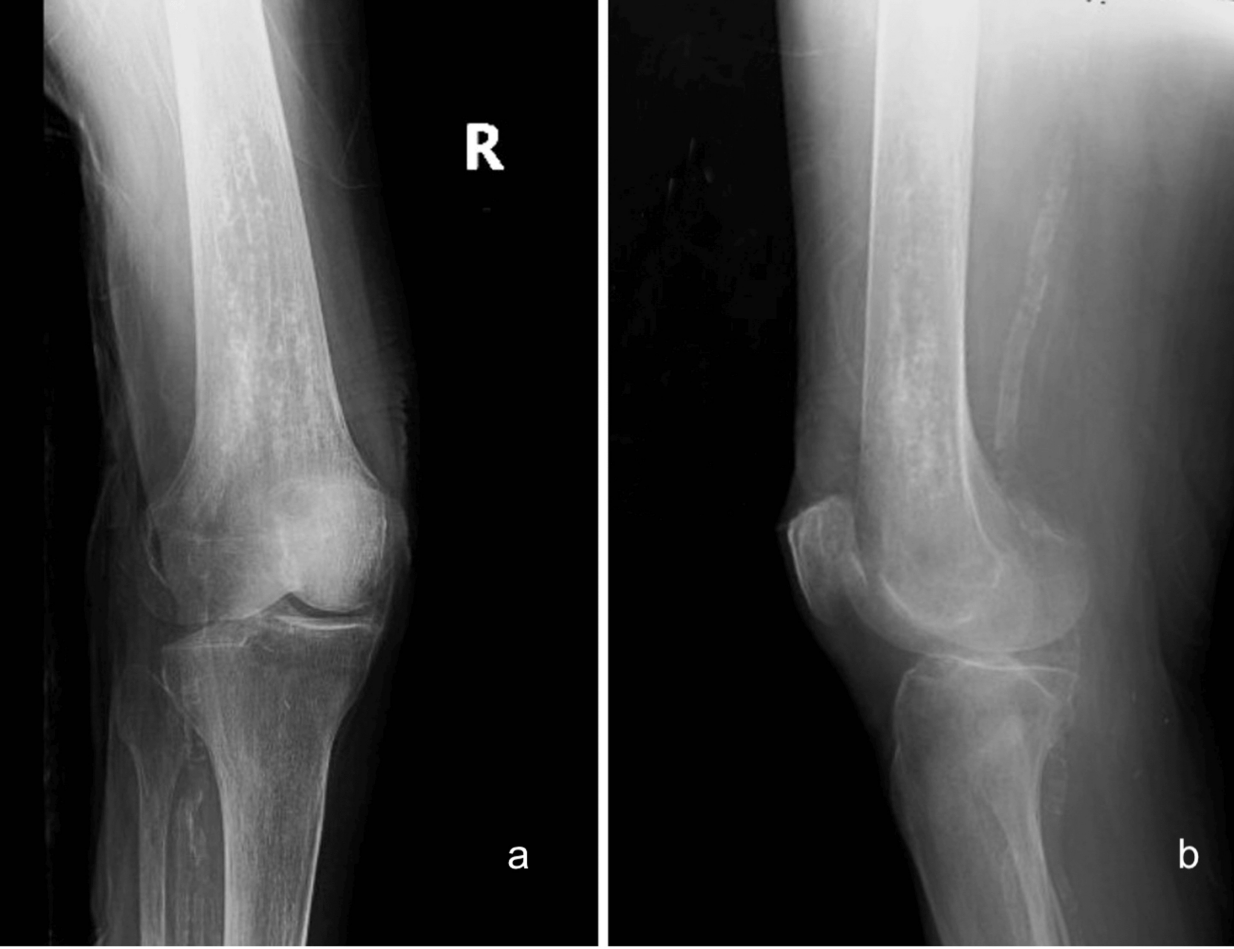
Anteroposterior (a) and lateral (b) radiographs of ipsilateral distal femur (right) showing intramedullary sclerotic lesion

Also, besides generalized osteopenia, there was a focal intra-medullary sclerotic lesion in the metadiaphyseal region of the distal femur, consistent with the appearance of a bone infarct evident in the radiographs (Figure 2).

**Figure 3:**
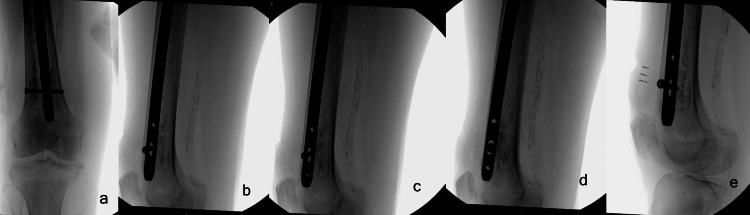
Fluoroscopic images showing anteriorly directed femoral nail abutting the cortex (a) Anteroposterior view; (b-e) lateral views.

As a standard of care, the decision was made to proceed with surgery, performing a closed reduction and internal fixation using a cephalomedullary nail through a trochanteric entry site. A Synthes TFN-ADVANCED® Proximal Femoral Nailing System (TFNA) (DePuy Synthes, West Chester, Pennsylvania) long nail (130 × 11 × 400 mm) was selected to safeguard the entire length of the femur against potential future fractures, given the patient's poor bone quality. The chosen nail has a radius of curvature of 100 cm. During implantation, the distal portion of the femoral nail passed anterior to the infarct, abutting the anterior femoral cortex on lateral fluoroscopy. Due to diffuse osteopenia, it was challenging to identify a specific area of cortical perforation (Figure 3).

**Figure 4:**
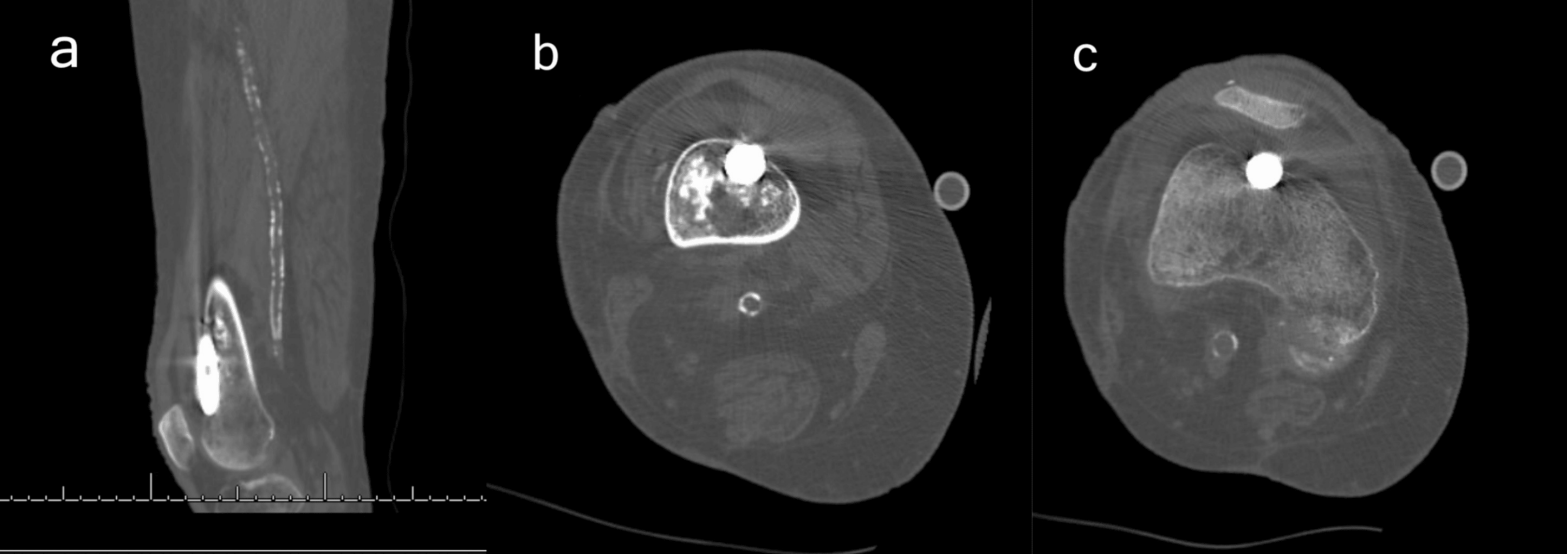
Postoperative CT images demonstrating anterior cortical breach (a) Sagittal cut; (b) coronal cut with sclerotic lesion and anterior cortical breach; (c) coronal cut with anterior cortical breach.

With the fracture adequately reduced and the rest of the hardware in a stable position, the decision was made to complete the surgery and obtain a computed tomography (CT) scan to evaluate the cortical perforation. Immediate postoperative CT imaging demonstrated that the distal nail tip had perforated the anterior femoral cortex near the proximal portion of the femoral trochlea (Figure 4).

**Figure 5:**
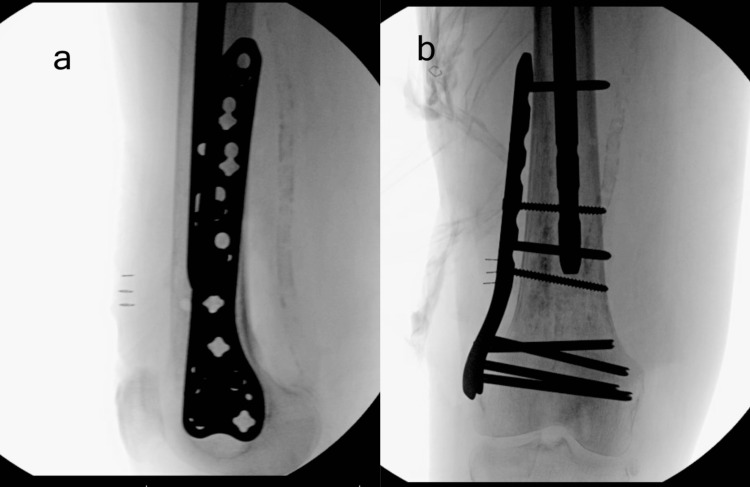
Revision surgery with exchange nailing supplemented with lateral locking plate (a) Lateral view; (b) anteroposterior view.

After a comprehensive discussion, the patient returned to the operating room for revision surgery. The previous hardware was removed and replaced with another intramedullary (IM) nail that was slightly shorter (Synthes TFNA 130 × 11 × 360 mm), stopping proximal to the cortical defect. Additionally, a six-hole distal femoral anatomic locking compression plate (Synthes DF-LCP) was placed through a separate lateral incision to span the cortical defect and augment the intramedullary fixation. The LCP was secured with multiple distal locking screws, while a combination of locking and cortical screws was used for proximal fixation, inserted around the intramedullary nail (Figure 5).

**Figure 6:**
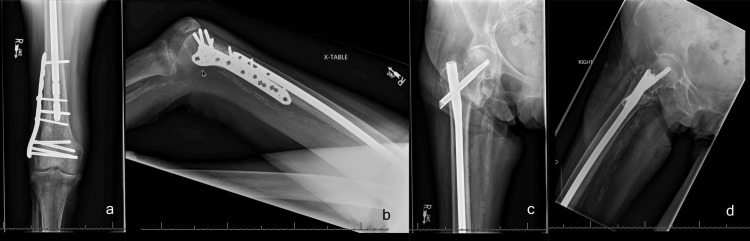
One-year postoperative X-rays (a) Anteroposterior view: distal; (b) lateral view: distal; (c) anteroposterior view: proximal; (d) lateral view: proximal.

All surgical wounds were irrigated and closed in a standard layered fashion. Postoperatively, the patient was allowed to bear weight as tolerated on the operative extremity with the assistance of physical therapy (PT) and was discharged in stable condition. The patient continued PT and made steady progress. At the 12-month follow-up after surgery, she was walking without pain at the fracture sites, and X-rays showed healing with maintained alignment and stable hardware position (Figure 6).

## Discussion

DFACP is a rare yet critical complication that can occur during antegrade IM nailing for proximal femoral fractures, with an overall incidence of 1% [4]. Given the potentially catastrophic consequences of DFACP, specifically peri-implant fracture, it is of paramount importance to be aware of, recognize, and address this complication as soon as it arises [5]. Mere impingement of the femoral nail on the anterior femoral cortex, even without a frank cortical breach, has been shown to result in higher rates of peri-implant fractures and should be avoided [6,7]. Our case report describes a comparable scenario resulting from an incidental intramedullary lesion, which, to the best of our knowledge, has not been previously described in the literature. The complication was promptly diagnosed using CT and effectively managed with revision surgery.

Risk factors

Historically, femoral bow and nail mismatch has been associated with DFACP. The average radius of curvature (ROC) of the femur is approximately 120 cm, whereas many older-generation antegrade IM nails had a larger ROC, ranging from 186 to 300 cm [8]. Newer-generation nails are designed to account for this anatomical variation, typically featuring lower ROCs, which reduce the risk of impingement and anterior cortex violation [9].

Several patient-specific risk factors for increased femoral bowing and the potential for DFACP have been identified in recent years [2,6]. These include short stature (<5'3"), female sex, poor cortical bone stock, and Asian or Hispanic ethnicity [10-14]. Proximal femur fractures are more commonly associated with DFACP than midshaft or distal femur fractures, although it remains unclear whether this complication is more frequent in subtrochanteric versus intertrochanteric femur fractures [5,15].

Preventive strategies

Meticulous attention must be paid intraoperatively to avoid anterior impingement of the IM nail on the distal femoral cortex. Preoperative imaging should include a full-length lateral view of the femur, which may alert the implant selection to patients with excessive sagittal bow. A more posterior start point for nail entry can result in anterior cortical impingement and/or perforation due to the creation of a more anterior trajectory as the nail passes through the narrow isthmic portion. Additionally, posterior positioning of the implant at the proximal femur necessitates extra external rotation for central lag screw placement, thereby increasing the implant's ROC [10,16-18]. Therefore, in patients with excessive femoral bow, a slightly anterior start point may help avoid anticipated distal anterior cortical impingement. It is equally important to closely monitor the guidewire position within the distal femur using dedicated true lateral fluoroscopic imaging. An inadvertent anterior positioning of the guidewire, as occurred in our case due to a distal femoral bony infarct, will result in eccentric reaming and anterior cortical breach. Every effort should be made to ensure central or even slightly posterior guidewire placement for concentric reaming of the distal femoral canal.

Various surgical techniques have been described to prevent DFACP. Yoon and Haidukewych proposed a technique for patients with capacious intramedullary canals, in which the femur was fully reamed with the smallest reamer, and subsequent larger reamers were used only up to the level of the canal isthmus to preserve medullary bone between the implant and the distal anterior cortex once the implant was seated [19]. El Beaino et al. utilized a blocking drill bit beneath the anterior cortex to prevent anterior migration of the guidewire and allow for central reaming of the canal [20]. Scolaro et al. recommended using a retrograde ball-tip guidewire directed from the knee for antegrade nail placement to prevent anterior abutment of the femoral cortex [21]. In cases where anterior cortical impingement persists, manual bending of the nail at its distal portion with a tabletop bender can be employed, albeit with the drawback of potentially weakening the nail [6].

Reaming through sclerotic bony lesions

Additional preoperative consideration is merited in the presence of intramedullary sclerotic lesions, such as the distal femoral bone infarct in our patient. These benign lesions are common in the knee (i.e., distal femur and proximal tibia) and the second most common anatomic site of involvement after the proximal femur [22-24]. To the best of our knowledge, there are no reports in the literature describing the risk of eccentric reaming when sclerotic intra-medullary lesions are present. Our patient’s sclerotic infarct in the distal femur lesion, combined with osteopenia, likely contributed to eccentric reaming and ultimately perforation of the anterior cortex during implant placement. We, therefore, recommend the judicious use of intraoperative fluoroscopy when reaming through sclerotic lesions and employing the appropriate preventive tactics as discussed above.

Recognizing cortical perforation

Intraoperative biplanar fluoroscopy is sufficient in most cases for avoiding anterior reaming and improper nail placement. However, this modality may be inadequate for detecting subtle perforations, particularly in patients with a large body habitus and/or poor bone quality. The trapezoidal anatomy of the distal femur and the depth of the trochlea can also confound fluoroscopic findings. Sarai et al. conducted a radiographic study comparing CT findings with fluoroscopy in 30 femur models with femoral nails placed in the anterior one-fifth of the distal femur, revealing that fluoroscopy missed 18 (60%) of anterior cortical perforations [25]. Although routine use of CT imaging is not necessary when evaluating intramedullary nail placement, we recommend utilizing this imaging modality in equivocal cases, either intraoperatively or postoperatively, to rule out cortical perforation.

## Conclusions

A higher index of suspicion for cortical perforation is recommended when a femoral nail is placed anteriorly in the presence of a distal femoral sclerotic lesion. Judicious use of multiplanar intraoperative fluoroscopy, combined with adjunct surgical techniques, is essential to prevent this complication. A CT scan is crucial for identifying subtle perforations, and if identified, revision surgery can help prevent periprosthetic fractures.
